# A Novel Method Using Fine Needle Aspiration from Tumor-Draining Lymph Nodes Could Enable the Discovery of New Prognostic Markers in Patients with Cutaneous Squamous Cell Carcinoma

**DOI:** 10.3390/cancers15133297

**Published:** 2023-06-22

**Authors:** Vilma Lagebro, Krzysztof Piersiala, Marianne Petro, Jan Lapins, Per Grybäck, Gregori Margolin, Susanna Kumlien Georén, Lars-Olaf Cardell

**Affiliations:** 1 Division of ENT Diseases, Department of Clinical Sciences, Intervention, and Technology, Karolinska Institutet, 171 77 Stockholm, Sweden; 2 Department of Otorhinolaryngology, Karolinska University Hospital, 171 76 Stockholm, Sweden; 3 Department of Medical Medicine, Unit of Dermatology, Karolinska Institutet, 171 77 Stockholm, Sweden; 4 Medical Radiation Physics and Nuclear Medicine, Karolinska University Hospital, 171 76 Stockholm, Sweden; 5 Department of Molecular Medicine and Surgery, Karolinska Institutet, 171 77 Stockholm, Sweden; 6 Medical Unit Head Neck, Lung and Skin Cancer, Karolinska University Hospital, 171 76 Stockholm, Sweden

**Keywords:** cutaneous squamous cell carcinoma, head and neck cancer, tumor-draining lymph node, prognostic factor, biomarker

## Abstract

**Simple Summary:**

Cutaneous squamous cell cancer is a common form of cancer. Caught early, it is usually curable. However, 3–5% develop into a metastatic form with a very poor prognosis. All efforts to find biomarkers, in blood or in the tumor itself, for early identification of this group of patients have so far failed. This study describes a novel method based on fine needle aspiration that enables the identification of lymphocyte markers in tumor-draining lymph nodes. By focusing on lymph nodes as a key organ for a well-functioning immune system, it might be possible to, early on, find biomarkers related to future metastatic development.

**Abstract:**

Cutaneous squamous cell cancer (cSCC) is the second most common form of skin cancer, characterized by abnormal, accelerated growth of squamous cells. When caught early, most cSCCs are curable. About 5 percent of the cSCC cases have advanced to such an extent, generally metastatic, that they are far more dangerous, with very poor prognosis and challenging to treat. All efforts to find biomarkers, in blood or in the tumor itself, for early identification of patients with a risk for metastasis have so far failed. The present study describes a novel method that enables the identification of lymphocyte markers in tumor-draining lymph nodes. Six patients with advanced cSCC were analyzed using a combination of a sentinel lymph node biopsy (SLNB) protocol, fine needle aspiration (FNA), and flow cytometry. Immunological results from the sentinel nodes were combined with corresponding data from peripheral blood and unfixed tumor tissues. The result demonstrates a striking difference between the subsets of T-cells from the three compartments. Our interpretation of this first pilot study is that the ability to follow specific immunological markers on lymphocytes in tumor-draining lymph nodes will enable the identification of novel prognostic biomarkers not detectable in material from blood and tumor tissues.

## 1. Introduction

Cutaneous squamous cell cancer (cSCC) is a common form of cancer with a high increase in incidence. The number of diagnosed cases in Sweden has almost doubled in the past 10 years [1], and the incidence rates are increasing globally [2]. The prognosis for cSCC is relatively good. However, approximately 3–5% of cSCC patients develop regional (resectable) or advanced (unresectable) metastasis in the lymph nodes [3,4,5]. Metastatic cSCC is considered lethal, with a mortality rate of over 70%, and for patients with advanced cSCC with distant metastases, the mortality rate is close to 90% [6,7]. To predict the risk for metastatic cSCC, it is of great importance to find prognostic factors for tumor progression. Today the known prognostic factors mainly focus on tumor size and tumor location [2,5]. However, Hillen et al. reported that most patients diagnosed with advanced cSCC had a primary tumor with a low T stage (Tis, T1, and T2) which implies that there must be other prognostic factors of importance than tumor features to determine progression [8]. Moreover, these risk factors derived from tumor features only give a snapshot of the disease progression at the time point of the excision of the tumor, which makes it difficult to monitor the progression of the disease over time [5,8]. It is, therefore, a priority to investigate new prognostic factors to improve the stratification of patients into groups based on high and low risk of progression. This could enable optimized patient care, prevent advanced cases of cSCC, save lives, and reduce costs.

A promising approach to finding prognostic factors could be to investigate the patient’s immune system and anticancer immune response [9,10,11,12]. The key organ for these functions is the lymph nodes (LN). The tumor-draining lymph nodes (TDLN) would be of special interest because they are the first LN or group of nodes that drain the tumor. The tumor is also most likely to metastasize to the TDLN if the cancer is progressing [12,13]. The TDLNs are essential for the anticancer immune response and are responsible for important immunological reactions such as antigen presentation, immune cell activation, priming, proliferation, and differentiation [12,14]. This implies that any disturbances in the TDLN could lead to severe consequences for the immune system and its ability to fight cancer progression. The TDLNs are investigated for staging purposes and sometimes resected using sentinel lymph node biopsy (SLNB), which is widely used in, e.g., melanoma [15] and breast cancer [16]. The use of SLNB in cSCC is currently under investigation [2]. In the past, the TDLN has been overlooked as a compartment worth investigating for its immunological factors. However, today the study of the immunological components of the TDLN is an emerging field, and the TDLN has been investigated in detail in, e.g., melanoma [17] and colorectal cancer [18]. 

The immunology of TDLN in cSCC patients has been investigated using immunohistochemistry (IHC) [19]. A drawback of this method is the lack of quantification and the need for surgically removed TDLNs. If the TDLN is removed, the compartment that would react to future immunological changes is lost [12]. Fine needle aspiration (FNA) could be used to follow any changes of immunological markers in the patients over time to circumvent these drawbacks because the FNA procedure does not resect the LN. Furthermore, flow cytometry on FNA, which is a suspension of lymph node cells and not an intact tissue, can be performed without prior tissue digestion, which would be necessary for resecting whole tissue. Avoiding the step with tissue digestion makes the quantification of cells easier, faster, and more reliable [20]. 

In this study, we aim to investigate if a novel method using a clinically established protocol for SLN detection combined with FNA and flow cytometry is a feasible method to detect lymphocyte markers in TDLNs from cSCC patients. We then aim to discover novel immunological prognostic tumor progression markers by comparing the lymphocyte markers in TDLN with the lymphocyte markers in peripheral blood and tumor tissue, respectively. 

## 2. Materials and Method

### 2.1. Patient Characteristics

Eligible patients enrolled in this study met the following inclusion criteria: (1) diagnosis of cSCC in the head or neck (HNcSCC); and (2) willingness to participate in the study. Exclusion criteria were as follows: (1) systemic autoimmune diseases; (2) synchronous second malignancies, hemo-lymphopoietic malignancies in the past; and (3) any other acute or chronic condition that could influence the immunological milieu in LNs. See Table S1: patient characteristics for other clinical patient characteristics. 

### 2.2. Lymphocyte Retrieval from the Sentinel Node Using Fine Needle Aspiration

Patients enrolled in the study went through a TDLN detection procedure before surgery to retrieve cells from the TDLN. The method has been described in our previous work [21]. To summarize, the tumor and adjacent tissue were injected with Technetium Tc 99m-labeled colloidal human serum albumin (Nanocoll^®^, GE-Healthcare, Chicago, IL, USA). A SPECT/CT was performed on the morning of the surgery. A handheld gamma probe was used to localize the sentinel nodes according to their radioactivity. When the sentinel nodes were detected, ultrasound was used to visualize the TDLN while cells were aspirated using FNA. The sample was transferred to 1 mL pre-chilled MACS Tissue Storage Solution (Miltenyi Biotec # 130-100-008, Bergisch Gladbach, Germany) and centrifuged at 500× *g* for 5 min. The cells were prepared for flow cytometry as stated below. 

### 2.3. Tumor Dissociation

After surgical excision, tumor samples were kept in pre-chilled MACS Tissue Storage Solution (Miltenyi Biotec # 130-100-008) and used within 1 h for further analysis. A Tumor Dissociation Kit (Miltenyi # 130-095-929) was used to dissociate the samples enzymatically and mechanically. After dissociation, cells were filtered through a 100 µm Cell Strainer (Corning # 352360, Glendale, AZ, USA). The cells were prepared for flow cytometry as stated below. 

### 2.4. PBMC Isolation

Fresh peripheral blood was collected in heparin tubes, and the peripheral blood mononuclear cells (PBMC) were separated with Ficoll-Hypaque (Cytiva #17144003, Marlborough, MA, USA) and Sepmate-50 tubes (STEMCELL Technologies #85460, Vancouver, BC, Canada) according to the manufacturer’s instructions. The cells were prepared for flow cytometry as stated below.

### 2.5. Flow Cytometry

PBMC, TDLN, and tumor cell pellets were resuspended in PBS. The cell suspensions were incubated with Fc-block (BD #565388, Stockholm, Sweden) and live/dead fixable Far Red dead cell stain (BD #L10120) for 5 min at room temperature. Samples were then stained with an antibody panel (CD45, CD3, CD4, CD8, CD20, CD127, CD25, CD69, HLA-DR, CTLA-4, and PD-1, see Table S2: antibody panels for flow cytometry analysis) for 20 min at room temperature followed by a wash with PBS, 500× *g* for 5 min. Cells were resuspended in PBS with 1% paraformaldehyde (HistoLab #02178, Seoul, Republic of Korea) and analyzed on a flow cytometer (BD Biosciences, LSR FORTESSA). Analysis of the flow cytometry data was performed with FlowJo version 10.8.1 (LLC, Ashland, OR, USA). See Figure S1: gating strategy of lymphocytes from PBMC, TDLN, and tumor. The flow cytometry panel was chosen to differentiate lymphocytes (CD45^+^), B cells (CD20^+^), T cells (CD3^+^), and various T cell subsets such as cytotoxic T cells (CD8^+^) and T helper cells (CD4^+^). Moreover, T regulatory cells (Tregs) can be detected using CD127 and CD25. To further investigate these cell types and subsets, activations markers (CD69 and HLA-DR) and checkpoint inhibitor (CPI) molecules (CTLA-4 and PD-1) were detected as well. 

### 2.6. t-SNE and FlowSOM

FACS 3.0 FCS-files from each compartment (PBMC, TDLN, and tumor) from 3 patients were imported into FlowJo software version 10.8.1 (LLC, Ashland, OR, USA). The FlowJo plugin FlowAI (v.2.3.1) [22] was used to exclude anomalies. Live cells were gated, followed by doublet exclusion. Next, CD45^+^ cells were gated and normalized to 1093 events using the downsample plugin (v.3.3.1). The samples were put together using the concatenation tool in FlowJo. A t-SNE was performed to reduce the dimensions of the multiparameter data (FlowJo v.10.8.1). The following parameters were used to create the t-SNE: iterations = 1000, perplexity = 30, learning rate (eta) = 699, KNN algorithm exact (vantage point tree) and gradient algorithm Barnes-Hut. Clusters of phenotypically related cells were then detected by the FlowJo plugin FlowSOM (v.3.0.18) [23]. FlowSOM was run with the following parameters: number of meta clusters = 8, SOM grid size 10 × 10, plot channels all as pie charts, node scale 100%, background meta clusters, and the following markers, CD3, CD4, CD8, CD20, CD127, CD25, CD69, HLA-DR, CTLA-4, and PD-1. The clusters were then visualized using the FlowJo plugin ClusterExplorer (v.1.6.6). The same phenotype markers were used in FlowSOM and ClusterExplorer. 

### 2.7. Statistical Analysis

Statistical analyses were performed with GraphPad Prism version 9.4.1 (GraphPad Software, La Jolla, CA, USA). All data are shown as mean ± SD. A *p*-value of <0.05 was considered statistically significant (* *p* < 0.05, ** *p* < 0.01 and *** *p* < 0.001). One-way ANOVA and Dunn’s multiple comparisons test were performed, and depending on if the data was paired or not, the Friedman test or Kruskal–Wallis test was performed. The data was not set to be normally distributed. 

### 2.8. Ethics Statement

All procedures performed in this study involving human participants were approved by the Swedish Ethical Review Authority and followed the ethical standards and guidelines of the institutional and/or national research committee and the Declaration of Helsinki. Informed and signed consent was obtained from all individual participants included in the study. Ethics committee approval: 2019-03518.

## 3. Results

In total, six patients were included in this study. Samples were taken from three compartments, PBMC, TDLN, and tumor. The samples were analyzed with flow cytometry using a surface marker panel. The developed method was feasible and resulted in flow cytometry data that was analyzed with gating and cluster analysis. In contrast to the proportions of cells, the MFI data does not show any significant differences between the compartments. Hence, the MFI data is not included below.

### 3.1. TDLN Accumulate CD4^+^ T Cells While PBMC Accumulate CD8^+^ T Cells

**Figure 1:**
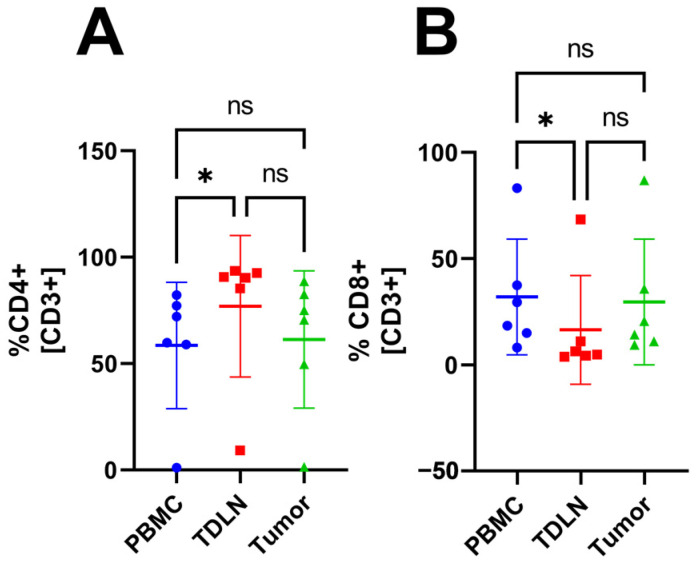
The proportion of T cell subsets in PBMC, TDLN, and tumor. The proportion of CD4^+^ (**A**) and CD8^+^ (**B**). Significant results indicated by * *p* < 0.05 and not significant results indicated by ns.

Gating analysis of the flow cytometry data reveals a significantly lower proportion of CD4^+^ T-cells in PBMC compared with TDLN (58.54 ± 29.67% vs. 76.93 ± 33.30%, respectively) see Figure 1A. In addition, a significantly higher proportion of CD8^+^ T-cells are seen in PBMC compared with TDLN (31.94 ± 27.20% vs. 16.45 ± 25.58%, respectively), see Figure 1B. Some subsets could not be gated in every compartment due to a low cell number.

### 3.2. Accumulation of Activated T Cells in TDLN and Tumor Compared to PBMC

**Figure 2:**
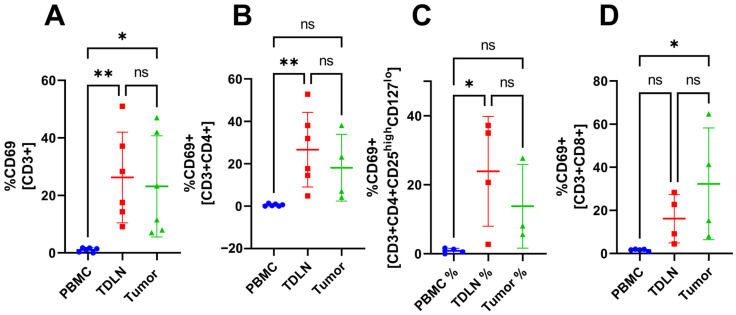
The proportion of T cell subsets in PBMC, TDLN, and tumor. The proportion of CD3^+^CD69^+^ (**A**), CD3^+^CD4^+^CD69^+^ (**B**), CD3^+^CD4^+^Treg^+^CD69^+^ (**C**), and CD3^+^CD8^+^CD69^+^ (**D**). Significant results are indicated by * *p* < 0.05 and ** *p* < 0.01. Not significant results are indicated by ns.

Gating analysis of the flow cytometry data reveals a significant accumulation of CD3^+^CD69^+^ T-cells in TDLN and tumor compared with PBMC (26.22 ± 15.80%, 23.13 ± 17.61%, 1.06 ± 0.73%, respectively), see Figure 2A. Furthermore, a significant accumulation of CD3^+^CD4^+^CD69^+^ is seen in TDLN compared with PBMC (26.64 ± 17.54% vs. 0.55 ± 0.56%, respectively), see Figure 2B. A significant accumulation of CD3^+^CD4^+^Treg^+^CD69^+^ is also seen in TDLN compared with PBMC (23.90 ± 15.91% vs. 0.87 ± 0.73%, respectively) see Figure 2C. A significant accumulation of CD3^+^CD8^+^CD69^+^ is shown in the tumor compared with PBMC (32.31 ± 25.91% vs. 1.62 ± 0.44%, respectively), see Figure 2D. 

### 3.3. Accumulation of Activated CD3^+^ T Cells and Tregs in Tumor Compared to PBMC and TDLN

**Figure 3:**
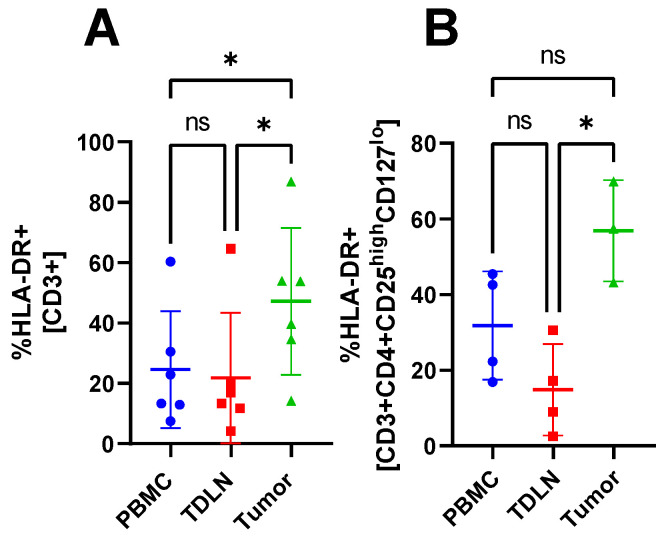
The proportion of T cell subsets in PBMC, TDLN, and tumor. Proportion of CD3^+^HLA-DR^+^ (**A**) and CD3^+^CD4^+^Treg^+^HLA-DR^+^ (**B**). Significant results indicated by * *p* < 0.05 and not significant results indicated by ns.

Gating analysis of the flow cytometry data reveals a significant accumulation of CD3^+^HLA-DR^+^ cells in the tumor compared with PBMC and TDLN (47.17 ± 24.33%, vs. 24.57 ± 19.33% and 21.78 ± 21.65%, respectively) see Figure 3A. Furthermore, CD3^+^CD4^+^Treg^+^HLA-DR^+^ cells are significantly accumulated in the tumor compared with TDLN (56.83 ± 13.36% vs. 14.84 ± 12.10%, respectively), see Figure 3B.

### 3.4. Accumulation of CTLA-4^+^ T Cell Subsets in the Tumor Compared to TDLN and PBMC

**Figure 4:**
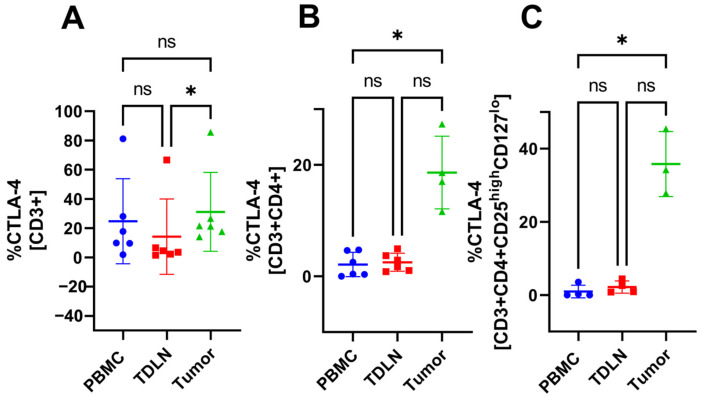
The proportion of T cell subsets in PBMC, TDLN, and tumor. Proportion of CD3^+^CTLA-4^+^ (**A**), CD3^+^CD4^+^CTLA-4^+^ (**B**), and CD3^+^CD4^+^Treg^+^CTLA-4^+^ (**C**). Significant results indicated by * *p* < 0.05 and not significant results indicated by ns.

Gating analysis of the flow cytometry data reveals a significant accumulation of CD3^+^CTLA-4^+^ cells in the tumor compared with TDLN (31.15 ± 27.01% vs. 14.19 ± 25.74%, respectively) see Figure 4A. Furthermore, CD3^+^CD4^+^CTLA-4^+^ cells are accumulated in the tumor compared with PBMC (18.63 ± 6.51% vs. 2.12 ± 2.19%, respectively), see Figure 4B. Lastly, CD3^+^CD4^+^Treg^+^CTLA-4^+^ cells are significantly accumulated in the tumor compared with PBMC (35.8 ± 8.91% vs. 0.94 ± 1.72%, respectively), see Figure 4C. 

### 3.5. High-Dimensional Cluster Analysis Revealed Different Cluster Compositions in the Three Compartments

**Figure 5:**
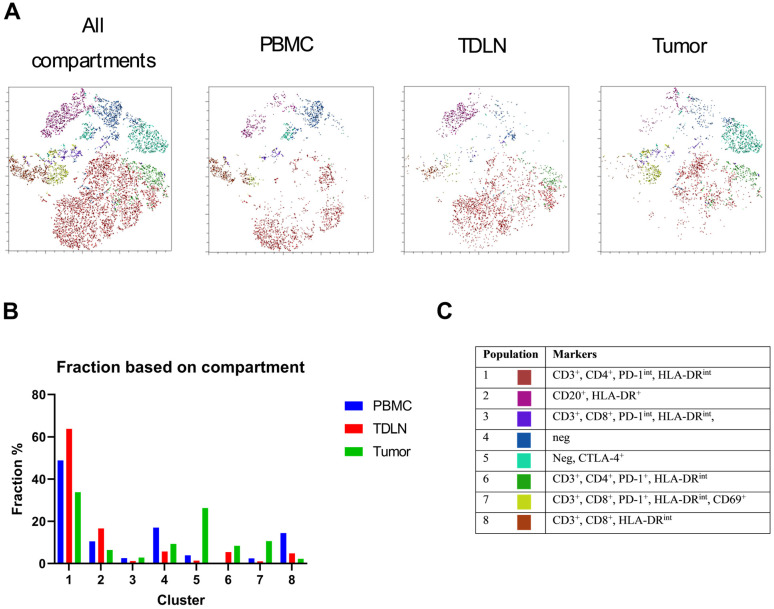
(**A**) Comparison of FlowSOM-derived cluster patterns of CD45^+^ cells from patients with cSCC. Cluster analysis was performed with three patients. The t-SNE plots with color-coded clusters were derived using FlowSOM clustering, illustrating the most prominent clusters for all the compartments and the compartments PBMC, TDLN, and tumor. (**B**). Comparison of the distribution of each cluster in the three compartments generated with FlowSOM. The FlowSOM was made with CD45^+^ cells from PBMC, TDLN, and tumors from patients with cSCC. (**C**). Table summarizing expression of CD3, CD4, CD8, CD20, PD-1, CTLA4 CD69^+^, and HLA-DR in different FlowSOM derived clusters.

Flow cytometry data from PBMC, TDLN, and tumors from 3 patients were concatenated, and a cluster analysis was performed to further analyze the data. The method is described in more detail in Section 2. The analysis resulted in t-SNE plots with FlowSOM-derived clusters. Eight unique clusters are identified, and their distribution and localization can be seen in Figure 5A, where the t-SNE plots are divided based on the compartments. The division based on compartments reveals distinct differences between the three compartments, both in the t-SNE plots and numerically in Figure 5B. The phenotype for each cluster is analyzed and is presented in Figure 5C. The Tregs are not differentially expressed in the clusters and are therefore not included among the markers in Figure 5C.

## 4. Discussion

This is the first descriptive study presenting a novel method using a combination of an SLNB-based protocol with FNA and flow cytometry. The method worked sufficiently to investigate lymphocyte markers from TDLN in cSCC patients. We described the lymphocytes in the three essential compartments for an anticancer immune response: PBMC, TDLN, and tumor. 

The MFI data did not show any significant differences between the compartments indicating that the cells express similar amounts of activation markers (CD69 and HLA-DR) and CPI molecules (CTLA-4 and PD-1). Furthermore, the number of CD20^+^ cells was too low to be analyzed. The CPI molecule PD-1 was included in the flow cytometry panel as well but did not show any significant difference in proportion between the three compartments. However, the flow cytometry data revealed a significant proportion difference between PBMC and the tumor for multiple T cell subsets. For example, the tumor had a higher proportion of activated T cells and Tregs expressing CTLA-4. A previous study [24] has shown a larger proportion of Tregs in the tumor compared with PBMC, which was confirmed in this study. However, they also report a difference in the proportion of CD4^+^ cells which cannot be seen in our study. Perhaps a larger *n* could have confirmed this because they had *n* = 17 compared with our *n* = 6. However, because the aim of our study was to test the feasibility of the method, we have left this question for future studies. A comparison of the proportions of CD3^+^, CD4^+^, CD8^+^, and CD4^+^Treg^+^ cells in PBMC, TDLN, and tumor revealed that TDLN was skewed toward either PBMC or the tumor, depending on which subset one looks at. The TDLN is significantly different from PBMC in some T cell subsets and significantly different from the tumor in other subsets, see Figure 1, Figure 2, Figure 3 and Figure 4. The fact that TDLN at times, is more like PBMC and sometimes more comparable to the tumor signifies its role as an intermediate key compartment. These results also emphasized the importance of investigating the TDLN instead of an exclusive focus on PBMC and the tumor micro-environment when conducting studies on the anticancer immune response. 

To further analyze our data, we conducted a cluster analysis. The cluster analysis was performed with three patients. This was due to one patient being analyzed on an earlier flow cytometry compensation and thus having different marker expression levels, which could not be normalized for. Furthermore, two patients were excluded due to their tumor samples having less than 100 CD45^+^ cells which were determined not to be statistically significant. The two patients were thus excluded because the remaining samples (PBMC and TDLN) would bias the cluster analysis if they were to be included. The cluster analysis showed that the compartments had different proportions of the clusters, indicating their dissimilarities and confirming the gated data. Most cells in all three compartments were found in cluster 1, which was most abundant in TDLN compared with PBMC and tumor, see Figure 4B. This cluster consists of CD3^+^, CD4^+^, PD-1^int^, and HLA-DR^int^ cells, see Figure 4C. A second cluster also included CD4 T cells, cluster 6, but only made up below 10% of the total cell population, see Figure 4B. If one were to merge clusters 1 and 6, TDLN would still have the highest proportion of CD4 cells. This has been confirmed in our previous work on gated flow cytometry data in oral squamous cell carcinoma [25]. Cluster 2 represents the B cells, and this cluster was also most abundant in TDLN compared with PBMC and tumor, see Figure 4B,C. CD8^+^ T cells are represented in three clusters, clusters 3, 7, and 8. Both clusters 3 and 7 express PD-1 and are more abundant in the tumor compared with PBMC and TDLN, while cluster 8, which did not express PD-1, was the least abundant in the tumor compared with PBMC and TDLN, see Figure 4B,C. This has also been confirmed in our previous work on gated flow cytometry data in oral squamous cell carcinoma [25]. Moreover, Cluster 5 contained a phenotype that was neither CD3^+^ nor CD20^+^ and expressed CTLA-4, see Figure 4B,C. Even though the cell phenotype could not be elucidated, this cluster, together with 3 and 7, demonstrates the immunosuppressive environment in the tumor. Cluster 4 contained negative cells for all markers and must, like cluster 5, have another phenotype that was not included in the flow cytometry panel, see Figure 4B,C. These clusters contain CD45^+^ cells and no debris because CD45^+^ cells were gated before the cluster analysis. Hence, these clusters could contain NK cells, which are CD45^+^ but not positive for CD3 or CD20. Interestingly, no Treg phenotype was seen in the cluster analysis, even though the gating analysis revealed that each compartment had around 10% CD4^+^Treg^+^ cells. As mentioned above, perhaps a large *n* would have elucidated these cells in the cluster analysis, but because the focus of the study was to investigate the performance of the new method, this question is left for future work. Future studies should probably also include FoxP3 in the flow cytometry panel. FoxP3 is an intra-cellular marker detecting Tregs, which could aid in the identification of these cells. FoxP3 was not included in the present panel because our focus was primarily on the feasibility of the method. 

A limitation of this type of method is the need for trained medical personnel that can perform FNA and sentinel node detection on the patients. However, if the hospital can perform SLNB, this method does not need any additional infrastructure or personnel with specific training. Further studies are needed to make this method available to patients. Firstly, the impact multiple FNA has on the lymph node and its immunology must be investigated deeper to determine the method’s biological limitations. However, our research group has conducted multiple fine needle aspirations in studies of intra-lymphatic immunotherapy for allergies [26,27]. In these studies, the lymph nodes were punctured three times during a 5-year period without any signs of adverse immunological effects. Secondly, to find prognostic markers, larger longitudinal studies with a larger *n* and a larger flow cytometry panel would be needed. 

In this study, we have demonstrated that our novel method works as intended. The cells received from the TDLN via FNA are abundant and possible to analyze using flow cytometry and subsequent cluster analysis. The most important advantage of using this method is the possibility of following the immunological landscape and how it changes over time. This gives far more power to the investigation of metastasis prognostic markers than using resected samples that only give an immunological snapshot of a one-time point during potential tumor progression. In the future, this method could have dual use, both as a method to find prognostic markers and as a subsequent way of monitoring these markers.

## 5. Conclusions

Even though the number of patients included is limited, and the study therefore must be considered as a pilot, the presented data clearly demonstrates the feasibility of using flow cytometric analysis of fine needle aspirated samples to find prognostic markers in patients with cutaneous squamous cell carcinomas. It is tempting to suggest that TDLN as an intermediate key compartment could lay the basis of future prognostic markers for advanced cSCC. However, further investigation of multiple fine needle aspirations will be needed to establish the full potential of this novel method.

If the results of studies with larger sample sizes and multiple follow-ups turn out to be successful, the new prognostic biomarkers could be used to improve the stratification of patients into low- and high-risk groups of cancer progression. Patients with a higher risk of progression would undergo a more rigorous and frequent follow-up scheme. Finally, this study could further strengthen the role of flow cytometry analysis in the clinical assessment and staging of cSCC patients.

## Data Availability

The data presented in this study are available upon request from the corresponding author.

## Supplementary Materials

The following supporting information can be downloaded at: https://www.mdpi.com/article/10.3390/cancers15133297/s1, Figure S1: Gating strategy of lymphocytes from PBMC, TDLN and tumor. (A). Live cells were gated followed by single cells and CD45^+^ cells. (B). CD20^+^ cells and CD3^+^ cells were gated from CD45^+^ cells. CD4^+^ and CD8^+^ cells were gated from the cells that were CD3^+^. (C). Tregs were gated from the CD4^+^ cells. Table S1: Patient characteristics. Table S2: Antibody panels for flow cytometry analysis.
