# Supplementary material for: A Novel Method Using Fine Needle Aspiration from Tumor-Draining Lymph Nodes Could Enable the Discovery of New Prognostic Markers in Patients with Cutaneous Squamous Cell Carcinoma
Source: Cancers (Basel). 2023 Jun 22;15(13):3297. doi: 10.3390/cancers15133297 (PMC10340690; doi:10.3390/cancers15133297)
Supplement: Supplementary file 1 [file cancers-15-03297-s001.zip › cancers-2379502-supplementary.pdf]

Supplementary Materials

Table S1. Patient characteristics.

Patient	Sex	Age at diagnosis	ICD10	Localisation	pTNM classification			Oncological treatment			Follow-up			PAD
					pT	pN	pM	CTH	RTH	BTRT	Relapse	Time to relapse (months)	Time without relapse (months)	
1.	M	81	C442S	Ytteröra	pT1	NO	0	yes	no		no	-	17	Medelhögt diff
2.	M	84	C442S	Ytteröra	pT2	NO	0	yes	no		no	-	18	Medelhögt diff
3.	M	85	C440	Underläpp	pT2	N0	0	no	yes	yes	no	-	17	Medelhögt diff
4.	M	81	C443S	Kind	pT2	N0	0	yes	no		no	-	18	Medelhögt diff
5.	M	77	C442S	Ytteröra	pT2	N0	0	yes	no		no	-	17	Medelhögt diff
6.	F	85	C443 C770	Skalpen	PT2	N1	0	yes	no		no	-	18	Medelhögt diff

Table S2. Antibody panels for flow cytometry analysis.

Antigen	Fluorochrome	Clone	Cat #	Antibody supplier
CD20	BUV395	2H7	563782	BD
CD45	BUV805	HI30	612891	BD
CLTA4 (CD152)	BV421	BNI3	562743	BD
CD8	BV510	RPA-T8	563256	BD
CD127	BV711	HIL-7R-M21	563165	BD
CD25	FITC/ BB515	M-A251	555431	BD
PD1 (CD279)	PE	EH12.1	560795	BD
CD4	PerCp 5.5	13B8.2	A07752	Beckman Coulter
HLA-DR	PE-Cy7	G46-6	560651	BD
Live Dead Far Red	APC		L10120	Beckman Coulter
CD3	AF-700	SP34-2	557917	BD
CD69	APC-Cy7	FN50	557756	BD

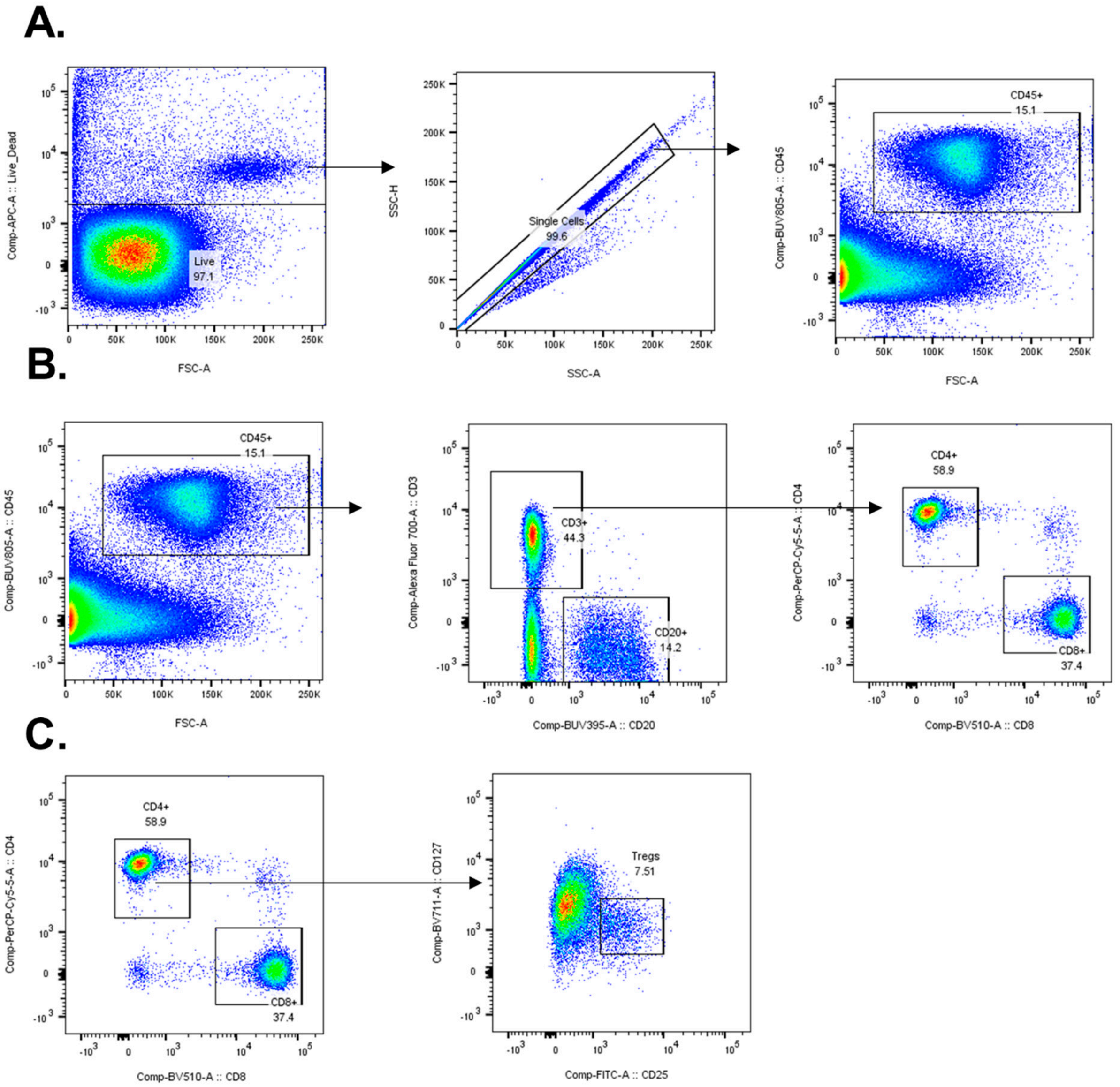

Figure S1. Gating strategy of lymphocytes from PBMC, TDLN and tumor. A. Live cells were gated followed by single cells and CD45+ cells. B. CD20 + cells and CD3+ cells were gated from CD45+ cells. CD4+ and CD8+ cells were gated from the cells that were CD3+. C. Tregs were gated from the CD4+ cells.
